# Switching Biological Therapies in Severe Asthma

**DOI:** 10.3390/ijms24119563

**Published:** 2023-05-31

**Authors:** Giulia Scioscia, Santi Nolasco, Raffaele Campisi, Carla Maria Irene Quarato, Cristiano Caruso, Corrado Pelaia, Andrea Portacci, Claudia Crimi

**Affiliations:** 1 Department of Medical and Surgical Sciences, University of Foggia, 71121 Foggia, Italy; giulia.scioscia@unifg.it; 2 Department of Clinical and Experimental Medicine, University of Catania, 95124 Catania, Italy; nolascos@hotmail.it (S.N.); raffaelemd@hotmail.it (R.C.); dott.claudiacrimi@gmail.com (C.C.); 3 Respiratory Medicine Unit, Policlinico “G. Rodolico—San Marco”, 95123 Catania, Italy; 4 Institute of Respiratory Diseases, Policlinico Foggia, 71121 Foggia, Italy; 5 Department of Medical and Surgical Sciences, Digestive Disease Center, Fondazione Policlinico A. Gemelli IRCCS, 00168 Rome, Italy; cristiano.caruso@policlinicogemelli.it; 6 Department of Health Sciences, University “Magna Graecia” of Catanzaro, 88100 Catanzaro, Italy; pelaia.corrado@gmail.com; 7 Department of Basic Medical Sciences, Neuroscience and Sense Organs, Section of Respiratory Disease, University “Aldo Moro” of Bari, 70121 Bari, Italy; a.portacci01@gmail.com

**Keywords:** severe asthma, biologics, randomized clinical trials, real-life studies, biomarkers, efficacy

## Abstract

Currently, three classes of monoclonal antibodies targeting type 2 inflammation pathways are available in Italy for the treatment of severe asthma: anti-IgE (Omalizumab), anti-IL-5/anti-IL-5Rα (Mepolizumab and Benralizumab), and anti-IL-4Rα (Dupilumab). Numerous randomized controlled trials (RCTs) and real-life studies have been conducted to define their efficacy and identify baseline patients’ characteristics potentially predictive of favorable outcomes. Switching to another monoclonal antibody is recommended in case of a lack of benefits. The aim of this work is to review the current knowledge on the impact of switching biological therapies in severe asthma as well as on predictors of treatment response or failure. Almost all of the information about switching from a previous monoclonal antibody to another comes from a real-life setting. In the available studies, the most frequent initial biologic was Omalizumab and patients who were switched because of suboptimal control with a previous biologic therapy were more likely to have a higher baseline blood eosinophil count and exacerbation rate despite OCS dependence. The choice of the most suitable treatment may be guided by the patient’s clinical history, biomarkers of endotype (mainly blood eosinophils and FeNO), and comorbidities (especially nasal polyposis). Due to overlapping eligibility, larger investigations characterizing the clinical profile of patients benefiting from switching to different monoclonal antibodies are needed.

## 1. Introduction 

Asthma is one of the most common chronic respiratory diseases worldwide, and it is characterized by airway inflammation, hyperactivity, and often reversible bronchial obstruction. Approximately 10% of patients have severe uncontrolled asthma despite high-dose inhaled corticosteroids and β2-adrenergic agonists [1,2], and they often experience unpredictable severe exacerbations requiring corticosteroid courses and/or hospitalizations [3]. In the last decade, clinicians have classified severe asthma according to type 2 inflammation level into “Type 2” and “Non-Type 2” subtypes [4]. Type 2 inflammation occurs in approximately 70% of severe asthma patients and is promoted by a broad network of hyper-expressed cytokines, namely IL-4, IL-5, IL-13, and several immune cells, including mastocytes, type-2 helper lymphocytes, type-2 innate lymphoid cells, basophils, and eosinophils.

Currently, three classes of monoclonal antibodies targeting type 2 inflammation pathways are available for treating severe asthma. They have shown efficacy in sparing OCS, reducing exacerbation rates, and improving asthma control and lung function [5,6]: anti-IgE (Omalizumab), anti-IL-5/Rα (Mepolizumab, Reslizumab, and Benralizumab), and anti-IL-4Rα (Dupilumab). Furthermore, new agents targeting epithelial cytokines known as “alarmins” (TSLP, IL-25, and IL-33) might also represent a valuable option for patients with unmet needs [7,8,9]. Despite the availability of different therapeutic options, around 10% of patients on biological treatment experience a suboptimal clinical response [10]. Current guidelines suggest switching to another monoclonal antibody in case of a lack of effectiveness [11]. In particular, the concept of “asthma remission” during treatment with biological drugs has recently gained great attention. The definition of remission in asthma includes various aspects of the disease, such as symptoms, exacerbations, lung function, and airway inflammation [12]. The non-achievement of remission could be a possible switching indicator. Nevertheless, there is a paucity of high-level evidence on how to choose the best initial biologic therapy, especially due to the lack of head-to-head comparisons among these drugs. Furthermore, controversies remain regarding the appropriate steps and timing for switching from one biologic to another [13]. 

We aim to provide a comprehensive review of the current knowledge on biologics for severe asthma and their switching patterns, as well as useful information on predictors of treatment response or failure.

### 1.1. Omalizumab

Omalizumab was the first monoclonal antibody to be approved as an add-on therapy for patients with severe persistent allergic asthma. It is a recombinant humanized IgG1 monoclonal antibody that inhibits the binding of free circulating IgE to the high-affinity IgE receptor (FcεRI) on the surface of both mast cells and basophils, thus limiting the degree of release of mediators of the allergic response. In Europe, it is indicated for severe allergic asthma patients over 6 years of age with a proven allergy to at least one perennial allergen and whose symptoms remain partially or totally uncontrolled despite receiving maximal medical therapy, including high doses of inhaled corticosteroids (ICSs), long-acting beta-agonists (LABAs), and eventually, other control agents. The serum total IgE should range from 30 to 1500 IU/mL in adults and children over 12 years and between 30 and 1300 IU/mL for children over 6 years. For patients over 12 years of age, reduced lung function (predicted FEV_1_ < 80%) is also required [14]. Omalizumab is administered at a dose of 150 to 375 mg by subcutaneous injection every 2 or 4 weeks. The dosage and frequency are calculated on the basis of body weight and total serum IgE levels. Furthermore, Omalizumab is also approved as an add-on therapy for chronic idiopathic urticaria who remain symptomatic despite H1 antihistamine treatment [15] and for severe chronic rhinosinusitis with nasal polyps with inadequate response to intranasal corticosteroids [16].

#### 1.1.1. Clinical Trials 

Since its approval, several randomized clinical trials (RCTs) have demonstrated the therapeutic efficacy of subcutaneously administered Omalizumab. The 28-week, randomized, double-blind INNOVATE study showed a statistically significant relative reduction of 26% in the rate of clinically significant asthma exacerbations (primary endpoint) compared with a placebo. Additionally, Omalizumab provided a favorable effect in a number of secondary outcomes over the placebo, including a reduction in severe exacerbations and improvement in asthma-related quality of life, morning peak expiratory flow, and asthma symptom scores [17]. In other RCTs, a significant reduction in asthma exacerbations was accompanied by a significant reduction in the hospitalization rate and the ICS requirement in patients receiving Omalizumab compared to those taking a placebo [18,19,20]. In the 48-week, randomized, double-blind EXTRA study, it seemed that Omalizumab treatment had a greater effect in reducing exacerbations in the subgroup of patients with high type 2 biomarkers at baseline, including FeNO, blood eosinophil count, and serum periostin [21]. However, other studies concluded that the drug was effective irrespective of the patients' baseline characteristics and biomarker levels [22]. In a pooled analysis of data from five RCTs, including 2236 patients with moderate to severe persistent allergic asthma receiving moderate to high-dose inhaled corticosteroids, improved clinical outcomes during treatment with Omalizumab were associated with decreased peripheral blood eosinophils, while worse clinical outcomes were associated with increased peripheral blood eosinophils [23]. This suggested that the efficacy of Omalizumab in responder patients may be due, at least in part, to an inhibitory effect on the release of type 2 cytokines (i.e., IL-4, IL-5, and IL-13) and eosinophil trafficking. The first evaluation of Omalizumab treatment efficacy is recommended after 16 weeks (i.e., 4 months) of treatment. After this observation period, it is possible to discontinue treatment due to lack of efficacy. 

Currently, data from RCTs assessing the impact of the Omalizumab switch in uncontrolled severe asthma patients are still limited. Chapman et al. [24], in a single-arm, multicenter, intention-to-treat Omalizumab switch to MepOlizumab (OSMO) clinical trial, demonstrated the effectiveness of mepolizumab administration in patients with a suboptimal response to Omalizumab treatment. The switch from Omalizumab to Mepolizumab 100 mg led to a substantial improvement in the Asthma Control Questionnaire (ACQ-5), above the minimum clinically important difference (MCID) of 0.5 points [25] and over a placebo effect control obtained from the main Mepolizumab metanalyses [26,27,28] after 32 weeks of treatment. Among secondary endpoints, 79% of patients improved their Saint George’s Respiratory Questionnaire (SGRQ) score above the MCID, showing a decrease in annual exacerbations rate and blood eosinophils count compared to the pre-switch period. FEV_1_ also improved over MCID after the Omalizumab to Mepolizumab switch, although that increase was considered below the authors’ expectations. No significant Mepolizumab-related adverse events were found, even during the running-in period, when an Omalizumab–Mepolizumab overlap was possible. 

A post hoc analysis conducted by Liu et al. [29] examined the response rate to the Omalizumab–Mepolizumab switch by performing several subgroup analyses based on blood eosinophil count, Omalizumab treatment regimen and duration, number of exacerbations in the previous year, use of OCS, comorbidities (nasal polyps, aspirin intolerance, gastroesophageal reflux disease), ACQ-5, SGRQ, body weight, and BMI quartiles. To define responders, the authors considered whether patients had a clinical benefit over MCID in ACQ-5 scores, pre-bronchodilator FEV_1_, SGRQ, and annualized rate of exacerbations requiring systemic corticosteroids or hospitalization. After the monoclonal antibody switch, 75% of patients were classified as responders according to ACQ-5 improvement, 78% for SGRQ, 69% for exacerbations, and 50% for FEV_1_. No significant differences were found in subgroup analyses, except for patients in the lowest ACQ-5 quartile, which showed less improvement in symptom control and quality of life. 

Magnan and colleagues showed similar findings in a post hoc analysis of MENSA and SIRIUS trials, considering the impact of Mepolizumab in patients with prior Omalizumab treatment [30]. Omalizumab treatment was discontinued in most enrolled patients due to a lack of efficacy (72% and 82%, respectively). However, clinical improvements and safety were similar in patients with and without previous Omalizumab administration, confirming that switching between these two monoclonal antibodies could be a reasonable choice in case of poor treatment response.

#### 1.1.2. Real-Life Studies

Omalizumab's effectiveness has been confirmed in several real-life studies [31]. This monoclonal antibody has shown an effective decrease in hospital admission rate, a decline in asthma exacerbations by 25%, an improvement in FEV_1_ by 250 mL after 12 months of treatment, and an improvement in asthma control test scores (ACT) and asthma-related quality of life questionnaire scores (AQLQ) [32,33]. 

Omalizumab was the first biologic agent for severe asthma without further options for clinicians. Therefore, switching to other biological therapies for personalized treatment was possible after introducing other monoclonal antibodies and improved phenotyping of severe asthma with the recognition of other therapeutic targets. Manzies-Gow et al. [34] considered all adults with severe asthma treated with biological therapy included in the International Severe Asthma Registry or the CHRONICLE study, observing that Omalizumab was the most common initial biologic treatment in 2015. In this study, the main reasons for switching from Omalizumab were lack of efficacy or potential adverse events. Patients undergoing the switch were more likely to have a higher baseline eosinophilic blood count and exacerbation rate, lower lung function, higher FeNO, greater healthcare resource utilization, more invasive ventilation episodes, emergency visits, and hospitalizations [34]. 

Several “real-world” studies have been performed in patients with poorly controlled severe eosinophilic asthma who were unsuccessfully treated with Omalizumab and switched to other biological drugs, such as Mepolizumab or Benralizumab. In these patients with a predominantly eosinophilic trait despite allergic ones, several asthma outcomes improved in terms of exacerbation rate, rescue medication need, asthma control, pulmonary function, IgE, FeNO, and eosinophilic counts [35,36,37,38]. These results were confirmed by a retrospective, single-centre study by O’Reilly et al. [39] in which patients remained suboptimally controlled despite Omalizumab and were switched to anti-IL-5 therapy. All these results highlight that switching from Omalizumab to other biologics for severe asthma is considered a valid therapeutic approach in order to improve asthma outcomes. Nonetheless, further prospective studies with well-defined switching criteria are required to identify better patients who may benefit from switching.

### 1.2. Mepolizumab 

Mepolizumab is a humanized IgG1/k monoclonal antibody that selectively binds IL-5 with high affinity, preventing its interaction with IL-5Rα, a receptor expressed by eosinophils, basophils, mast cells, and type-2 innate lymphoid cells [40]. This biologic is approved as an add-on maintenance treatment of severe uncontrolled asthma in patients aged over 6 years with an eosinophilic phenotype defined as peripheral blood eosinophils greater than or equal to 150 cells/μL at the initiation of treatment or greater than or equal to 300 cells/μL within the past 12 months. The recommended dose in adults is 100 mg, administered subcutaneously once every 4 weeks. Other indications include severe chronic rhinosinusitis with nasal polyps for whom therapy with systemic corticosteroids and/or surgery do not provide adequate disease control, relapsing–remitting or refractory eosinophilic granulomatosis with polyangiitis (EGPA), and inadequately controlled hypereosinophilic syndrome without an identifiable non-haematologic secondary cause. 

#### 1.2.1. Clinical Trials 

Timely clinical studies conducted in patients with mild to moderate persistent asthma, aside from the eosinophils count in blood and sputum, failed to show relevant changes in asthma symptoms and lung function [41,42,43]. Subsequently, monthly intravenous infusions of 750 mg of Mepolizumab were evaluated in small groups of subjects with uncontrolled severe eosinophilic asthma. In these patients, Mepolizumab effectively reduced eosinophils in blood and sputum and decreased asthma exacerbations and prednisone consumption while slightly enhancing FEV_1_ values [44,45]. In a phase IIb/III DREAM study, patients were randomly assigned to four groups receiving either a placebo or one of three doses of intravenous Mepolizumab (75, 250, or 750 mg) at intervals of 4 weeks in one year. At all dosages used, Mepolizumab effectively lowered the frequency of asthma exacerbations (up to 52%), regardless of IgE levels and atopic status [27]. In a phase III SIRIUS study, subcutaneous Mepolizumab 100 mg every 4 weeks for 20 weeks provided oral glucocorticoid sparing with a 50% reduction in prednisone dosage [46]. A larger phase III MENSA study showed that, in patients with an eosinophil count of at least 150 cells/μL in peripheral blood, administration of Mepolizumab every 4 weeks for 32 weeks at a dosage of 75 mg intravenously or 100 mg subcutaneously induced a significant reduction in asthma exacerbation rates of 47% and 53%, respectively [26]. In a phase IIIb MUSCA study, 100 mg of subcutaneous Mepolizumab induced an early and prolonged improvement in patients’ health-related quality of life score, with relevant reductions in asthma exacerbations [28].

Both COSMOS and COLUMBA, long-term open-label studies, confirmed the safety profile of Mepolizumab from previous clinical trials. Only 8% of patients in COLUMBA developed anti-drug antibodies, which in most cases were transient and showed no relationship between the frequency of AEs, or hypersensitivity reactions and the presence or absence of ADAs [47,48].

With regard to the comparative evaluation of the therapeutic effects of Mepolizumab and Omalizumab, a systematic literature review of clinical trials suggests that Mepolizumab is at least as effective as Omalizumab in preventing asthma exacerbations and improving lung function in patients with severe eosinophilic allergic asthma, eligible to receive both treatments [49]. A post hoc meta-analysis confirmed these data from two phase III studies (MENSA and MUSCA) performed in patients with Omalizumab prescription criteria. Indeed, reductions in clinically significant exacerbations with Mepolizumab versus placebo were similar in Omalizumab-eligible and ineligible patients (57% vs. 55%). Furthermore, FEV_1_, ACQ-5, and SGRQ scores improved with Mepolizumab versus placebo regardless of Omalizumab eligibility, Immunoglobulin-E levels, or atopic status, confirming that Mepolizumab 100 mg has a clinical benefit in patients with blood eosinophil counts ≥ 150 cells/μL, regardless of allergic characteristics [49]. 

#### 1.2.2. Real-Life Studies

Several real-world studies confirmed the efficacy of Mepolizumab in clinical practice [50,51,52] and analyzed the effect of switching, especially from Omalizumab. Bagnasco and colleagues performed a retrospective study enrolling 27 non-responder subjects to Omalizumab (unable to discontinue or reduce OCS, with two or more exacerbations/year during treatment, or at least one hospitalization) switched to Mepolizumab after 1 month of washout. After 12 months of Mepolizumab, the mean annual exacerbation rate decreased by 81%, with a parallel increase in FEV_1_ and ACT scores, which exceeded the threshold of 20 in the overall cohort, indicative of well-controlled asthma [35]. In another retrospective study from Italy, 41 consecutive patients with severe allergic eosinophilic asthma, with previous unsuccessful anti-IgE treatment, were switched to Mepolizumab without a washout period. Omalizumab failure was defined as a lack of effectiveness (frequent exacerbations and/or uncontrolled symptoms) after at least 12 months of treatment. After 1 year of Mepolizumab, patients experienced an 83% decrease in the annual exacerbation rate, an increment of ACT score, an increase in pre-bronchodilator FEV_1_, and a reduction of blood eosinophils while also lowering the percentage of patients who were dependent on corticosteroids, from 46% with Omalizumab to 5% after 12 months of Mepolizumab [36]. Furthermore, switching to Mepolizumab, in the case of Omalizumab failure, led to a reduction in lost working days and a slight increase in economic costs related to biological treatment, outweighed by the reduction in annual exacerbations and the limitation of adverse events related to prolonged OCS consumption [37]. These data suggest that Mepolizumab provides clinically important benefits for patients with overlapping allergic and eosinophilic phenotypes with high blood eosinophil counts. Indeed, in all the studies, the baseline value of blood eosinophils was >500 cells/μL, well above the 150 cells/μL threshold from clinical trials. In addition, the most reported comorbidity was nasal polyposis. In a post hoc analysis from clinical trials, patients with severe eosinophilic asthma and nasal polyposis seemed to experience greater benefits in terms of quality of life and exacerbation decrease with Mepolizumab, compared to those without nasal polyps [53]. An explanation for this finding is that the local generation of IL-5 within both upper and lower airways can result in higher circulating blood eosinophil levels, which are predictive biomarkers of a better response to Mepolizumab, suggesting that subjects with severe asthma, nasal polyps, and high blood eosinophils experience a better response to anti-IL-5 rather than to anti-IgE, irrespective of allergic status and IgE levels.

### 1.3. Benralizumab 

Benralizumab targets IL-5Rα in all cells expressing the receptor, including eosinophils, basophils, and mast cells. This results not only in the blocking of IL-5-mediated survival of these cells but also in a related increase in eosinophil apoptosis via antibody-dependent cell-mediated cytotoxicity (ADCC) induced by enhanced activation of the FcγRIIIa part of the IL-5Rα receptor of mature natural killer (NK) cells and macrophages, leading to a dramatic, almost complete decrease in peripheral eosinophils. Benralizumab is indicated as an add-on maintenance treatment in adult patients with severe eosinophilic asthma inadequately controlled despite high-dose inhaled corticosteroids plus long-acting β-agonists. The recommended dose is 30 mg by subcutaneous injection every 4 weeks for the first three doses and then every 8 weeks thereafter.

#### 1.3.1. Clinical Trials

The SIROCCO and CALIMA trials demonstrated that Benralizumab administered subcutaneously at a dose of 30 mg either every 4 weeks or every 8 weeks (with the first three doses administered every 4 weeks) was effective in significantly reducing the rate of exacerbations and in improving lung function and the asthma symptom score compared to placebo in severe uncontrolled asthma patients with an eosinophilic phenotype [54,55]. A pooled analysis of data from those two phase 3 studies revealed that the greatest improvement in the annual exacerbation rate was achieved in patients with high blood eosinophil thresholds (≥300 or ≥450 cells/μL) and a history of more frequent exacerbations (≥3) [56]. Moreover, Benralizumab was observed to be equally effective in both allergic and non-allergic severe eosinophilic asthma [57]. In the phase 3 ZONDA study, Benralizumab showed a clinically relevant benefit in reducing the use of OCS, as well as in reducing exacerbations when compared with placebo [58]. The long-term BORA and MELTEMI extension trials confirmed the safety and efficacy of Benralizumab for up to two and up to five consecutive years of treatment, respectively [59,60].

#### 1.3.2. Real-Life Studies

A large number of real-life studies have confirmed the clinical benefits of Benralizumab in improving respiratory function, asthma control, and quality of life, as well as in reducing the daily intake of OCS [61,62,63]. Benralizumab's effectiveness has been observed in both allergic and non-allergic individuals [64]. 

A study by Gómez-Bastero Fernández et al. [65] evaluated the efficacy of Benralizumab at 4 and 12 months in a group of 40 patients who had an inadequate response after therapy with Omalizumab (16 patients) or Mepolizumab (24 patients). After a switch to Benralizumab, there was an improvement not only in asthma control (as per ACT score) but also a drastic reduction in the number of severe exacerbations and, above all, in the use of OCS and hospital visits. 

In a recent post hoc analysis of the Italian multi-center observational ANANKE study by Caruso et al. [66], Benralizumab induced a reduction of over 90% in asthma exacerbations (including severe ones), also showing an important reduction in concomitant use of OCS (with nearly 50% of patients who were able to completely discontinue the use of OCS), as well as improvements in asthma control and lung function in both naïve patients and those previously treated with a biologic. 

Using a real-world approach, Pelaia et al. [38] recently evaluated the effectiveness of a therapeutic switch from Omalizumab to Benralizumab in 20 allergic patients with severe eosinophilic asthma. These patients experienced inadequate asthma control during anti-IgE treatment with Omalizumab. Indeed, although Omalizumab was able to significantly decrease asthma exacerbations, these were not completely prevented by anti-IgE therapy after at least one year of treatment. On the other hand, after 12 months, Benralizumab was able to dramatically reduce exacerbations of severe eosinophilic asthma, not only when compared to the 12 months preceding Omalizumab therapy but also with respect to the effects of this latter biologic drug. By nearly zeroing disease exacerbations, Benralizumab was capable of completely avoiding asthma-related hospitalizations. Regarding the Omalizumab period, Benralizumab induced a valuable trend towards a further decrement in prednisone intake. An OCS-sparing action made it possible for 13 (76%) out of 17 patients to interrupt OCS consumption. When compared to this remarkable result, Omalizumab induced OCS withdrawal in only four subjects (24%). Differently from Omalizumab treatment, the noticeable improvement in asthma symptom control induced by Benralizumab led to a significant increase in ACT score (median value of 20) and an almost total abolition of rescue SABA inhalations. In addition to these clinical outcomes, Benralizumab also overcame Omalizumab by further enhancing pre-bronchodilator FEV_1_. Moreover, while Omalizumab left unchanged blood eosinophil counts, Benralizumab zeroed their numbers. This biological effect could reasonably explain the superiority of Benralizumab versus Omalizumab documented by our study. It is thus arguable that Omalizumab did not suppress airway eosinophilic infiltration, which was otherwise effectively abrogated by Benralizumab. Therefore, the persistence of refractory eosinophilic asthma accounted for the unsuccessful effect of Omalizumab. On the contrary, the very effective anti-eosinophilic therapy provided by IL-5 receptor blockage, mediated by Benralizumab, likely underlies the therapeutic success afforded by this monoclonal antibody. The therapeutic differences between Omalizumab and Benralizumab extended from the bronchial tree to the upper airways, as shown by the further decrease of SNOT-22 score, noticed after Omalizumab replacement with Benralizumab.

Regarding Mepolizumab towards Benralizumab switching, Numata et al. [67] reported that Benralizumab induced only a slight but not significant improvement in several clinical and functional parameters monitored in 11 asthmatic patients undergoing such a therapeutic shift. However, other studies have clearly shown the superiority of Benralizumab versus Mepolizumab/Reslizumab within the population of severe asthmatics unresponsive or partially responsive to anti-IL-5 therapy. A recent retrospective multi-center, real-world investigation performed by Drick et al. [68] evaluated 60 patients who were shifted to Benralizumab among 665 asthmatic subjects receiving Mepolizumab or Reslizumab. This therapeutic switching was prompted by either an inadequate treatment response (50 patients) or the occurrence of adverse events (10 patients). When compared to a previous median 8 month-treatment with anti-IL-5 monoclonal antibodies, the subsequent median 5-month anti-IL-5Rα therapy elicited a progressive improvement in symptom control, OCS intake, and lung function. In addition, Benralizumab further potentiated blood eosinophil decrease, already induced by Mepolizumab or Reslizumab. Kavanagh et al. [69] evaluated a group of 33 asthmatic patients with an unsatisfactory response to Mepolizumab, defined as a failure to achieve either a ≥50% decrease in annualized exacerbation rate or ≥50% reduction in OCS maintenance therapy. They found a significant drop in the annualized exacerbation rate by 58% during Benralizumab treatment. Benralizumab also increased the percentage of patients who achieved a ≥50% OCS dose decrement and significantly improved symptom control and quality of life. Nevertheless, Benralizumab was not able to significantly increase FEV_1_ compared with baseline values. In 27 Spanish patients with severe eosinophilic asthma, the ORBE study was carried out by Martínez-Moragón et al. [70]. They recruited 24 subjects previously treated with Mepolizumab and three patients previously treated with Reslizumab prior to being shifted to Benralizumab. This therapeutic switching was induced by either an insufficient response or a previous intolerance to anti-IL-5 treatment. When considering the enrolled patients who received at least the first three doses of Benralizumab, significant improvements in ACT score, annualized asthma exacerbation rate, and OCS intake occurred. No significant FEV_1_ increase was detected; however, nine patients experienced a relevant FEV_1_ increment of more than 200 mL. Moreover, regarding asthma-related healthcare resources, Benralizumab also decreased the requirements for non-scheduled primary care and specialist visits. 

The above real-world observations suggest that the superiority of Benralizumab versus Mepolizumab/Reslizumab, referring to patients with severe eosinophilic asthma partially unresponsive to anti-IL-5 therapy, depends on the peculiar mechanisms of Benralizumab action. Indeed, differently from IL-5 inhibition provided by Mepolizumab/Reslizumab, Benralizumab not only blocks the α subunit of the IL-5 receptor but also directly induces eosinophil apoptosis through antibody-dependent cell-mediated cytotoxicity. Hence, it can be inferred that when compared to Mepolizumab and Reslizumab, Benralizumab can exert a more effective anti-eosinophil therapeutic action. 

### 1.4. Dupilumab 

Dupilumab is a monoclonal antibody blocking the α-subunit of the IL-4 receptor (IL-4R), of which two forms are distinguished. Type I IL-4R, expressed on the surface of blood cells, is composed of the link of the IL-4α subunit with the γ-chain subunit. Type II IL-4R, expressed by cells of the bronchial epithelium and skin, comes from the union between the IL-4α subunit together with the α1 subunit of the IL-13 receptor, forming a heterodimer capable of binding both IL-4 and IL-13 [71,72]. IL-4 is a driving factor for T cell differentiation towards the Th2 subtype and induces the production of T2-associated cytokines and chemokines, such as IL-5, IL-9, IL-13, and eotaxins. It promotes the class switching of B-cell immunoglobulin towards IgE and IgG4 [72,73]. In addition, this interleukin induces the overexpression of vascular cell adhesion molecule-1 (VCAM-1), which is involved in eosinophil recruitment from blood circulation to the lungs through the interaction with α4-integrin [74]. Along with IL-4, IL-13 stimulates the production of eosinophil-promoting factors, including IL-5 and eotaxin-3, and it is a potent inductor of the inducible isoform of the enzyme nitric oxide synthase (iNOS) in the airway epithelium. The amount of NO released by the bronchial epithelium can be non-invasively measured in a breath test, and FeNO is considered a T2 biomarker. Furthermore, IL-13 plays a crucial role in airways hyperactivity and tissue remodelling, stimulating mucus hypersecretion by goblet cells, smooth muscle alterations, and type-1 collagen deposition from fibroblasts [75,76,77]. Given its blocking action on the IL-4α subunit, Dupilumab is capable of inhibiting both IL-4 and IL-13 signalling. This monoclonal antibody has been approved as an add-on maintenance treatment for severe asthma with T2 inflammation characterized by raised blood eosinophils (a blood eosinophil count of 150 cells/μL or more) and/or raised FeNO (FeNO of 20 parts per billion or more). Additionally, it can be employed for the treatment of different T2 inflammatory diseases, including atopic dermatitis [78] and nasal polyps [79]. For severe asthma, the typical loading dose of Dupilumab is 400 mg, followed by a maintenance dose of 200 mg every other week, administered as a subcutaneous injection. For patients with OCS-dependent severe asthma and/or with comorbidities, such as atopic dermatitis or chronic rhinosinusitis with nasal polyposis, an initial dose of 600 mg is recommended, followed by 300 mg every other week. 

#### 1.4.1. Clinical Trials

Pivotal phase 2 clinical trials demonstrated the effectiveness of Dupilumab in improving asthma control, quality of life, and lung function, reducing the asthma exacerbation rate [80,81,82]. 

Dupilumab treatment has been demonstrated to decrease levels of T2 biomarkers, such as FE_NO_ and serum IgE, while blood eosinophil levels have been shown to remain unchanged or even increase [80]. However, the phase 2 QUEST study revealed that the efficacy of Dupilumab was highest in patients characterized by elevated levels of systemic eosinophils and F_E_NO (i.e., ≥150 cells/µL and ≥25 ppb, respectively) [83]. The larger phase 3 QUEST trial confirmed the encouraging clinical outcomes obtained in the previous phase 2 studies [84]. In addition, the phase 3 VENTURE study demonstrated no loss of asthma control, a reduction in asthma exacerbation rate, and an improvement in lung function, despite the reduction in oral corticosteroid (OCSs) use in patients treated with Dupilumab compared with a placebo group [85]. The study by Corren et al. [86] revealed similar favourable clinical outcomes after Dupilumab treatment in allergic and non-allergic moderate-to-severe asthmatics.

#### 1.4.2. Real-Life Studies

Currently available real-life studies confirmed the efficacy data of the RCTs mentioned above. In a retrospective multicenter study on 64 uncontrolled severe asthma patients, Dupin et al. [87] observed that Dupilumab significantly improved asthma control and lung function, reducing oral steroids use and exacerbations rate at 3, 6, and 12 months after treatment. Furthermore, real-life studies have shown to be useful to better understand whether specific clinical characteristics and/or biomarkers make patients more responsive to treatment and how to translate this information into clinical practice. In this regard, Carpagnano et al. [88] reported that introducing FeNO levels in the evaluation of type-2 severe asthma might further help identify patients eligible for Dupilumab. Pelaia et al. [89] suggested that Dupilumab could be used as a valuable add-on biological therapy with rapid onset of action in both allergic and non-allergic asthmatic patients with nasal polyposis. 

Alongside this evidence of Dupilumab's effectiveness in the treatment of severe asthma, only limited data exist about switching from a previous monoclonal antibody therapy to an anti-IL-4/13 biologic, and all the available information comes from real-life studies. In 2021, Mümmler et al. [90] retrospectively analyzed 38 severe asthma patients that were switched to Dupilumab from a previous anti-IgE or anti-IL5/IL5Rα medication due to insufficient outcome. In total, 32 out of 38 patients, after 3 to 6 months of treatment with Dupilumab, experienced improved asthma control and lung function, decreased exacerbation rate, and FeNO and IgE levels. Patients with increased FeNO (≥25 ppb) during previous antibody therapy were more often responders to Dupilumab than patients with lower FeNO (<25 ppb). In an Italian real-life study analyzing one year of experience with Dupilumab, Campisi et al. [91] confirmed that this biological antibody represents a valid therapeutic option for non-responders to other biological therapies. Thus, out of a total of five patients who were switched from Omalizumab, Mepolizumab, or Benralizumab to Dupilumab due to a lack of therapeutic response, all subjects showed a reduction in the number of exacerbations and OCS use, as well as an improvement in the pre-bronchodilator FEV_1_% values and asthma control. In 2022, Numata et al. [92] observed that Dupilumab treatment effectively reduced exacerbations and OCS maintenance doses and improved asthma symptoms in patients with or without prior biological treatment. According to the findings of this study, the baseline blood eosinophil count (≥150 cells/µL before Dupilumab administration or ≥300 cells/µL prior to the use of any biologics) could be used to identify “super responders” to Dupilumab treatment. 

In a case series including four patients previously treated with an anti-IL-5 or anti-IL-5R biologic for OCS-dependent asthma, Eger et al. [93] showed that the switch to this anti-IL-4/IL-13 biologic, together with discontinued OCS use, may induce hypereosinophilia, with sudden deterioration of asthma, tissue infiltration by eosinophils, and EGPA-like symptoms, such as thromboembolic events. These authors warned clinicians to always consider that OCS-dependent severe asthma patients could have an underlying (ANCA-negative) EGPA (with high levels of blood eosinophils masked by OCS maintenance therapy) and suggested stopping Dupilumab and (re)starting anti-IL-5 or anti-IL-5R therapies if eosinophils rise to more than 1000 cells/mcL and asthma symptoms worsen. Eosinophilic complications may also occur despite an initial favorable response after switching from an anti-IL-5 or anti-IL-5R biologic to an anti-IL-4/IL-13 monoclonal antibody. This suggests that activated IL-5 and IL-4/IL-13 pathways can simultaneously contribute to airway inflammation in some cases of severe asthma. Indeed, during Dupilumab treatment, eosinophils can move from the bone marrow to the blood (as this process is mediated by IL-5), but they cannot reach the lungs, presumably because of the inhibition of IL-4/IL-13 signalling and the subsequent diminished expression of VCAM-1 adhesion molecules [74]. Briegel et al. [94] hypothesized that the combined blockage of the two pathways might result in optimal disease control in severe asthma patients, for those where an anti-IL-5 or anti-IL-5R treatment alone is insufficient, as well as in patients in which symptomatic hypereosinophilia occurs under Dupilumab treatment.

## 2. Conclusions

**Figure 1:**
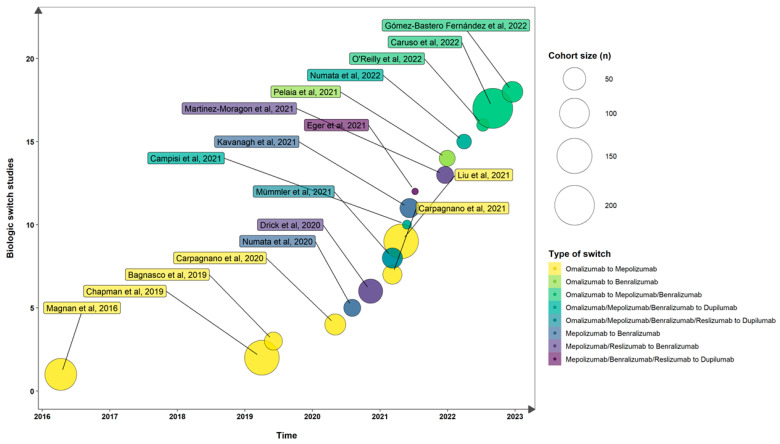
A chronological and cohort-size summary of the literature review [24,29,30,35,36,37,38,39,65,66,67,68,69,70,90,91,92,93].

Almost all of the information about switching from a previous monoclonal antibody to another in severe asthma come from small real-life experiences (Figure 1). 

The main results of available studies that investigated switching between biologics in severe asthma are summarized in Table 1. 

Overlapping eligibility in monoclonal antibody switching deserves more attention, particularly the exploration of data from the national and international severe asthma registries and consensus recommendations due to its major clinical and pharmaco-economical relevance. 

Asthma is a chronic respiratory disease, affecting patients for their whole life. As age increases, changes can take place in the immune system, as well as structural alterations, that are thought to be associated with the release from the allergic mechanism leading to a change of endotype and with the pathophysiology of late onset asthma. In this regard, the magnitude of the effects of Omalizumab seems to be lower in older patients than in younger ones, while anti-IL5 and anti-IL4/IL13 therapy appear to show even more pronounced effects in late onset disease and in asthmatic patients over 65 years old [95]. A limitation of this review is certainly the lack of information on switching to Tezepelumab, due to this drug is not yet available on the market.

In conclusion, further studies characterizing the clinical profile of patients benefiting the most from biologics in severe asthma are warranted to avoid multiple switches between biologics.

## Figures and Tables

**Table 1:** Summary of clinical studies focused on switching between biological therapies in severe asthma.

Study	Study Type	Switch	Study Population	Results
Chapman et al. [24], 2019 (OSMO study)	Clinical trial	From Omalizumab to Mepolizumab (observation: 36 weeks)	138 patients affected by allergic eosinophilic asthma with a suboptimal response to Omalizumab were switched to Mepolizumab	Clinically significant improvements in asthma control, health status, and exacerbation rate, with no tolerability issues reported.
Liu et al. [29], 2021	Post hoc analysis of OSMO study	From Omalizumab to Mepolizumab (observation: 36 weeks)	Subgroup analyses of patients included in the OSMO study (*n* = 138) to evaluate the influence of baseline characteristics (blood eosinophil count, comorbidities, exacerbation history, oral corticosteroid use, ACQ-5 and SGRQ scores, body mass index) on the results of the switch from Omalizumab to Mepolitzumab	Improvements were observed regardless of baseline characteristics.
Magnan et al. [30], 2016	Post hoc analyses of MENSA and SIRIUS studies	From Omalizumab to Mepolizumab (observation: 32 weeks in MENSA and 20 weeks in SIRIUS)	Post hoc analyses to assess the effectiveness of Mepolizumab in patients with severe eosinophilic asthma previously treated with Omalizumab included in the MENSA (75 patients, 13%) and SIRIUS (45 patients, 33%) studies	Patients responded positively to Mepolizumab regardless of prior use of Omalizumab.
Bagnasco et al. [35], 2019	Real-life study	From Omalizumab to Mepolizumab (observation: 1 year)	27 patients with severe allergic eosinophilic asthma were switched to Mepolizumab due to lack of control despite Omalizumab treatment	Significant reduction in mean yearly exacerbations and mean dose of OCS (daily mg of prednisone) and significant improvement in mean FEV1 and mean ACT score.
Carpagnano et al. [36], 2020	Real-life study	From Omalizumab to Mepolizumab (observation: 1 year)	41 patients with severe allergic eosinophilic asthma, with previous unsuccessful anti-IgE treatment, were switched to Mepolizumab without a washout period.	Increase in ACT score and in pre-bronchodilator FEV_1_, with a reduction in exacerbations and blood eosinophils. The percentage of patients who were dependent on corticosteroids also lowered.
Carpagnano et al. [37], 2021	Real-life study	From Omalizumab to Mepolizumab (observation: 1 year)	33 patients with severe eosinophilic asthma undergoing a switch to Mepolizumab because they were not optimally controlled by Omalizumab	Decrease in annual exacerbations and adverse events related to prolonged OCS consumption with a consequent reduction in the number of lost working days.
Pelaia et al. [38], 2021	Real-life study	From Omalizumab to Benralizumab (observation: 1 year)	20 patients with severe persistent allergic and eosinophilic asthma, uncontrolled despite the add-on biological treatment with Omalizumab, and thus switched to Benralizumab.	Significant improvements in asthma exacerbation rate, rescue medication need, ACT score, FEV_1_ and blood eosinophil count.
O’Reilly et al. [39], 2022	Real-life study	From Omalizumab to anti-IL5 or anti-IL5Rα therapy (observation: 1 year)	10 patients switched to an anti-IL-5 therapy (6 patients switched to Benralizumab and 4 to Mepolizumab) due to suboptimal control despite Omalizumab	Significant reductions in community exacerbation rate and serum eosinophil count and a significant improvement in FEV1 from baseline.
Gómez-Bastero Fernández et al. [65], 2022	Real-life study	From Omalizumab to anti-IL5 or anti-IL5Rα therapy (observation: 4 and 12 months)	40 patients switched from Omalizumab (*n* = 16) or Mepolizumab (*n* = 24) to Benralizumab due to lack of response (30 cases), adverse effects (9 cases) or patient request (1 case)	Significant decrease in the number of exacerbations, visits to the emergency department, and corticosteroid cycles. ACT score also improved. However, no significant improvement in lung function was observed.
Caruso et al. [66], 2022	Post hoc analysis of the ANANKE study (real-life study)	From Omalizumab or Mepolizumab to Benralizumab (observation: 16, 24 and 48 weeks)	147 biologic-naïve and 58 biologic-experienced asthma patients (34 Omalizumab, 19 Mepolizumab, and 5 Omalizumab-Mepolizumab) were observed after Benralizumab introduction	Similar reductions in exacerbations (>90% in both groups), OCS use (≥49% reduction in OCS dosage), ACT improvement and lung function were observed within the two groups.
Numata et al. [67], 2020	Real-life study	From Mepolizumab to Benralizumab (observation: 4 months)	Among 24 patients treated with Mepolizumab, 11 had directly switched to Benralizumab due to a lack of asthma control	Slightly improvement in some parameters, but without significant differences.
Drick et al. [68], 2020	Real-life study	From anti-IL5 therapy to anti-IL-5Rα (observation: 5 months)	Among 665 asthmatic subjects receiving anti-IL5 treatment, 60 patients (12 receiving Reslizumab and 48 receiving Mepolizumab) were switched to Benralizumab	Progressive improvement in symptom control, OCS intake and lung function.
Kavanagh et al. [69], 2021	Real-life study	From Mepolizumab to Benralizumab (observation: 48 weeks)	33 asthmatic patients with an unsatisfactory response to Mepolizumab underwent a switch to Benralizumab	Reduction in the annualized exacerbation rate by 58%, significant improvement in symptom control and quality of life, and increase in the percentage of patients who achieved a ≥50% OCS dose decrement. However, no significant increase in FEV_1_ compared with baseline values.
Martínez-Moragón et al. [70], 2021 (ORBE study)	Real-life study	From anti-IL5 therapy to anti-IL5Rα (observation: until a mean of 5 months between the first and the last Benralizumab treatment dosage)	24 subjects previously treated with Mepolizumab and 3 patients previously treated with Reslizumab were shifted to Benralizumab due to lack of efficacy	Significant improvements in ACT score, annualized asthma exacerbation rate, and OCS intake occurred. No significant FEV_1_ increase was detected.
Mümmler et al. [90], 2021	Real-life study	From anti-IgE or anti-IL5/IL5Rα therapies to Dupilumab (observation: from 3 to 6 months)	38 severe asthma patients were switched to Dupilumab from a previous anti-IgE or anti-IL5/IL5Rα medication due to insufficient outcome	32 out of 38 patients, improved asthma control and lung function, decreased exacerbation rate, and F_E_NO and IgE levels. Patients with increased F_E_NO (≥25 ppb) during previous antibody therapy were more often responders to Dupilumab than patients with lower F_E_NO (<25 ppb).
Campisi et al. [91], 2021	Real-life study	From Omalizumab, Mepolizumab or Benralizumab to Dupilumab (observation: 12 months)	5 patients were switched from Omalizumab, Mepolizumab, or Benralizumab to Dupilumab due to a lack of therapeutic response	Reduction in the number of exacerbations and OCS use, as well as improvement in pre-bronchodilator FEV_1_% values and asthma control in all the subjects.
Numata et al. [92], 2022	Real-life study	From Omalizumab, Mepolizumab or Benralizumab to Dupilumab (observation: mean follow-up of 12.6 months)	10 patients received Dupilumab as the first biologic, and 16 switched to Dupilumab from other biologics	Reduction in exacerbations and OCS maintenance doses and improvement in asthma symptoms regardless of prior biologic treatment. The baseline blood eosinophil count (≥150 cells/µL before Dupilumab administration or ≥300 cells/µL prior to the use of any biologics) seemed to identify “super responders” to Dupilumab.
Eger et al. [93], 2021	Case series	From anti-IL-5 or anti IL-5Rα biologics to Dupilumab (observation: variable case by case)	4 patients previously treated with an anti-IL-5 or anti-IL-5R biologic for OCS-dependent asthma who were switched to Dupilumab	The switch to Dupilumab, together with discontinued OCS use, induced hypereosinophilia, with sudden deterioration of asthma, tissue infiltration by eosinophils, and EGPA-like symptoms, such as thromboembolic events.

## Data Availability

The authors confirm that the data supporting the findings of this study are available within the article.
